# Retinopathy of prematurity: inflammation, choroidal degeneration, and novel promising therapeutic strategies

**DOI:** 10.1186/s12974-017-0943-1

**Published:** 2017-08-22

**Authors:** José Carlos Rivera, Mari Holm, Dordi Austeng, Tora Sund Morken, Tianwei (Ellen) Zhou, Alexandra Beaudry-Richard, Estefania Marin Sierra, Olaf Dammann, Sylvain Chemtob

**Affiliations:** 1 Department of Pediatrics, Ophthalmology and Pharmacology, Centre Hospitalier Universitaire Sainte-Justine Research Center, 3175, Chemin Côte Ste-Catherine, Montréal, Québec Canada; 2 Department of Ophthalmology, Maisonneuve-Rosemont Hospital Research Center, University of Montréal, Montréal, Québec Canada; 3 Department of Laboratory Medicine, Children’s and Women’s Health, Norwegian University of Science and Technology (NTNU), Trondheim, Norway; 4 Department of Ophthalmology, Trondheim University Hospital, Trondheim, Norway; 5 Department of Neuromedicine and Movement Science, Norwegian University of Science and Technology (NTNU), Trondheim, Norway; 6 Departament of Public Health Community Medicine, Tufts University School of Medicine, Boston, MA USA; 7 Perinatal Neuroepidemiology Unit, Hannover Medical School, Hannover, Germany

**Keywords:** Inflammation, Neuron-derived factors, Choroidal degeneration, Retinopathy of prematurity

## Abstract

Retinopathy of prematurity (ROP) is an important cause of childhood blindness globally, and the incidence is rising. The disease is characterized by initial arrested retinal vascularization followed by neovascularization and ensuing retinal detachment causing permanent visual loss. Although neovascularization can be effectively treated via retinal laser ablation, it is unknown which children are at risk of entering this vision-threatening phase of the disease. Laser ablation may itself induce visual field deficits, and there is therefore a need to identify targets for novel and less destructive treatments of ROP. Inflammation is considered a key contributor to the pathogenesis of ROP. A large proportion of preterm infants with ROP will have residual visual loss linked to loss of photoreceptor (PR) and the integrity of the retinal pigment epithelium (RPE) in the macular region. Recent studies using animal models of ROP suggest that choroidal degeneration may be associated with a loss of integrity of the outer retina, a phenomenon so far largely undescribed in ROP pathogenesis. In this review, we highlight inflammatory and neuron-derived factors related to ROP progression, as well, potential targets for new treatment strategies. We also introduce choroidal degeneration as a significant cause of residual visual loss following ROP. We propose that ROP should no longer be considered an inner retinal vasculopathy only, but also a disease of choroidal degeneration affecting both retinal pigment epithelium and photoreceptor integrity.

## Background

Retinopathy of prematurity (ROP) represents an important cause of childhood blindness worldwide [1, 2]. In high-income countries, ROP-associated blindness incidence has been reported to be lower than 10% of extremely preterm born children; however, in low- and particularly middle-income countries, the incidence is greater than 40% with increasing survival of infants born preterm and limited fundoscopic follow-up evaluation [3–5].

ROP is considered a multifactorial disease, and its pathogenesis has been extensively studied in humans and in several animal models. In premature infants, the development of ROP proceeds with an initial phase of retinal microvascular degeneration [6, 7] associated with an arrest in progressive vascularization of the peripheral retina. These ﻿va﻿scular changes result in retinal ischemia which predisposes to abnormal intravitreal neovascularization leading to its most significant sequelae retinal detachment and permanent visual loss. Even though pathological neovascularization in ROP may be prevented with treatment limiting tissue ischemia (laser ablation of the retina) and/or hypersecretion of VEGF (intravitreal injection of anti-VEGF), still a clear understanding of the mechanisms implicated in the progression of ROP from phase 1 to phase 2 is needed to develop new therapeutic alternatives. So far, several risk factors in the initial phase of ROP have been discussed [8, 9]. For instance other than prematurity, growth restriction is in addition to hyperoxia, an established risk-factor for ROP development [10, 11]. According to this, the WINROP tool (weight, insulin-like growth factor I, neonatal, retinopathy of prematurity) based on neonatal growth and measurements of levels of insulin-like growth factor-1 (IGF-1) has recently been developed as a prognostic marker [12]. On the other hand, it is known that premature neonates are susceptible to infection because of immature immune system. Inflammation has been shown to play an important role in the development of normal and pathological angiogenesis in the retina [13–16]. Interestingly, in the last 5 years, a series of epidemiological studies have supported the hypothesis that neonatal inflammation is a key modulator in the development and progression of ROP [17–19]. In the ELGAN study cohort**,** inflammatory stimuli such as bacteria in the placenta [20] and late bacteremia [21] were risk factors for developing ROP. Moreover, systemic inflammation in neonates has been shown to perturb retinal vessel development and to induce pathological features of ROP in animal models [22, 23]. Inflammatory factors such as cytokines, chemokines, hypoxia-inducible factors, hormones, nitric oxide, growth factors, or inflammatory cells such as leucocytes, monocytes, or macrophages/microglia are implicated in the control of angiogenesis and/or play a detrimental role in the developing vasculature [24, 25]. Furthermore, the influence of inflammation in the regulation of neuron-derived signaling molecules that causes endothelial cell injury during ROP has recently been highlighted. Notably, the beneficial or detrimental effect of all these components may depend on the time of action, duration, concentrations, and target tissue.

The retina is essentially an outgrowth of the brain where neural and vascular tissue develops in close proximity during fetal and neonatal life [26]. It is therefore conceivable that pathological processes that occur in the developing retina also can occur in the brain. An example of this is the augmented inflammatory response associated with retinal and preterm brain injury during hypoxia-ischemia [27, 28]. Therefore, in addition to the risk of a poor visual outcome, infants with ROP are at increased risk of dysfunctions associated with non-visual disabilities such as brain damage [29], physical and cognitive impairment at 5 years [30, 31], below-grade-level academic performance at 8 years [32], and lower health-related quality of life at 10 years of age [33]. In fact, approximately 55% of children with ROP sustain long-term neurodevelopmental disabilities [34]. These studies suggest a shared etiology of visual and non-visual developmental disabilities in preterm born children. Consequently, expanding the knowledge of ROP pathogenesis has the potential to contribute to preventing both the pathological vascularization and the risk of retinal detachment in ROP, as well as the complications and diseases that are associated with ROP.

An enigma on long-term outcome following ROP has been the residual visual loss that may occur in patients regardless of regression of neovascular changes. Indeed, there are indications that ROP affects the late-maturing central retina with long-term deficits in photoreceptor-functioning [35, 36]. The choroid supplies the central outer retina with oxygen and nutrients, and deficits in choroidal maturation could participate in visual deficits following ROP [35]. Knowledge on how choroidal degeneration influences the integrity of the retinal pigment epithelium (RPE) and photoreceptor layers might be important in understanding the long-term damages following ROP, such as residual visual loss.

The present review addresses established concepts as well as emerging evidence implicating inflammation in the pathogenesis of ROP, the detection of choroidal degeneration and possible consequences in ROP, and some promising therapeutic strategies for this disorder.

### Inflammatory factors in ROP

The role of inflammation in ROP has been poorly investigated. Recent evidences suggested that prenatal, perinatal, and postnatal inflammation might contribute to a gradual increase in the risk for ROP [17]. Cytokines and chemokines are small proteins secreted by immune cells that play a central role in distinct inflammatory processes including the progression of ROP. Current evidences about the role of these inflammatory factors in the development of ROP will be discussed in the following section.

#### Cytokines

Both the fetus and the preterm newborn are capable of mounting a significant inflammatory response [37], often linked to maternal infection transmitted to the preterm infant [38, 39]. The inflammatory response is a highly regulated process, where an elevated concentration of one cytokine often is associated with elevated levels of others (21). Cytokines such as IL-1β, TNF-α, and IL-6 act as primary initiators of inflammation following infection or tissue damage [40], although both pro- and anti-inflammatory properties have been observed [41]. These initiators of inflammation can mediate cytokine receptor activation, which leads to downstream upregulation of effector molecules such as chemokines (e.g., IL-8, RANTES) and adhesion molecules (e.g., ICAM-1) [42]. Interestingly, IL-1β and TNF-α produced by retinal microglia cells following exposure to hypoxia have been associated with deleterious effects in the retina [43]. In the oxygen-induced retinopathy (OIR) model, IL-1β has been indirectly associated to retinal microvascular degeneration [44]; while in the choroid, it is directly responsible for the involution of the choroidal blood vessels that results in a hypoxic sub-retina and consequently loss of RPE and photoreceptor integrity [45].

IL-10 and IL-4, on the other hand, tend to be viewed as anti-inflammatory cytokines [41], capable of protecting the developing brain and possibly retina against ongoing inflammation. Although, a study showed that IL-10 can be implicated in promoting pathological angiogenesis in an OIR mice model [46], conversely, in vitro, IL-10 inhibits expression of TNF-α, MIP-1a, and RANTES in microglial cells [47]. Furthermore, in pregnant rats exposed to systemic inflammation, IL-10 treatment reduced the occurrence of brain damage in their newborn pups [48]. Infants with an IL-10 high-producer allele were less likely to have white matter damage on ultrasound and a trend (albeit not significant) towards a lower prevalence of severe ROP [49]. On the other hand, no differences in blood IL-4 concentration were found between infants with ROP and controls in cord blood [50] or vitreous [13]; however, elevated vitreous IL-4 concentrations have been detected in patients with diabetic retinopathy [51].

#### Chemokines

Chemokines induce chemotaxis and regulate movements of immune cells such as microglia to sites of inflammation. Chemokines of special interest for ROP pathophysiology are IL-8, RANTES, monocyte chemotactic protein 1 (MCP-1), and interferon-inducible T-cell α chemoattractant (I-TAC).

IL-8 is implicated in both inflammation and neovascularization pathological in the eye [52]. In humans, higher serum concentration of IL-8 right after birth was associated with later ROP deemed “in need of treatment” [53], whereas in rats, increased levels of an IL-8 homologue were detected during the peak of pathological neovascularization in a model of ROP [54].

RANTES plays an important role in innate immunity, which is particularly critical to the newborn until acquired immunity is developed. The role of RANTES in ROP development is not known; however, it has been found that the concentration of RANTES in the vitreous of eyes with vasoproliferative severe ROP tends to be low [13], and a lower serum level was detected in infants who later developed severe ROP [14, 55]. These data suggest that RANTES might play an important protective role during ROP, which warrants further investigation.

MCP-1 can attract a variety of immune cells and is expressed in a wide range of tissues including neurons, astrocytes, and activated microglia of the brain and neuroretina [56]. MCP-1 has the ability to disrupt the blood-brain barrier and is thought to contribute to the pathogenesis of multiple neurodegenerative diseases [56]. Preterm infants who later developed ROP tended to have higher cord serum concentrations of MCP-1 than both healthy preterm peers and infants born at term [57]. Among newborns who weighed < 1000 g, those who received/needed oxygen for more than 6 h had higher MCP-1 concentrations in blood collected on day 3 than their peers who received oxygen for a shorter amount of time (even after adjusting for potential confounders) [58]. In addition, several studies documented elevated levels of MCP-1 in the vitreous humor of patients with retinopathy [59, 60], and in animal models, MCP-1 was shown to be involved in the induction of the retinal neovascularization possibly by modulating or attracting macrophages/microglia during the ischemic phase of retinopathy [61, 62].

### Growth factors

Vascular endothelial growth factor (VEGF) and insulin-like growth factor-1 (IGF-1) have long been considered some of the main actors in the pathogenesis of ROP. However, recent research data suggest that neurotrophins, matrix metalloproteinases, HIFs, erythropoietin (EPO), placental growth factor (PlGF), basic fibroblast growth factors, angiopoietins, and thyroid-stimulating hormone (TSH) also play a significant role in the progression of ROP. Some of these evidences will be discussed below.

#### Neurotrophins: brain-derived neurotrophic factor and neurotropin 4

Neurotrophins belong to a family of growth factors that promote neuronal survival and differentiation both in the central and peripheral nervous systems [63]. Infants who developed severe ROP tended to have lower serum concentrations of neurotrophin-4 and brain-derived neurotrophic factor (BDNF) during the first 3 weeks of life than those who did not develop severe ROP [14]. In the same study cohort, specific gene variations of BDNF were associated with threshold ROP [64]. In a separate small study, 16 infants who developed ROP had lower BDNF concentrations than 7 who did not develop ROP on postnatal day 60 [65].

#### Metalloproteinases

The matrix metalloproteinases (MMP-1 and MMP-9), which are responsible for cleaving protein in the extracellular matrix, are important in fetal development, inflammatory responses, and angiogenesis [66, 67]. Factors that are implicated in preterm diseases such as reactive oxygen species (ROS), growth factors, and various cytokines initiate MMP transcription [68] thereby making metalloproteinases (MMPs) biomarkers of interest for ROP. Systemic inhibition of MMPs in mice reduced neovascularization in an OIR model, while increased concentrations of proteases in the retina (MMPs included) have been associated with the active phase of retinopathy [69].

#### HIF1-α and HIF2-α

HIFs are transcription factors that stimulate the release of a wide variety of growth factors such as members of the VEGF family, angiopoietins, and EPO. The α-subunits of HIF-1 and HIF-2 are of special interest in ROP pathogenesis since they are suppressed during hyperoxia (ROP phase 1) and upregulated by tissue hypoxia (ROP phase 2) [66, 70]. In mice, prevention of HIF-1α degradation in the hyperoxic phase 1 might prevent hyperoxia-related vessel loss [71], whereas drug-induced reduction of HIF-1 during the neovascular phase (phase 2) appears to reduce retinal neovascularization [72].

#### VEGF and its receptors (VEGFR 1 and 2)

VEGF is a sub-family of growth factors involved in the developing retinal vasculature. The active role of VEGF in ROP pathogenesis seems well established and is supported by both clinical studies [73, 74], and the benefits of intravitreal anti-VEGF injections during the neovascular phase of ROP [75]. While suppressed during the hyperoxic phase 1 in ROP development, high VEGF levels in phase 2 disturb normal vascularization. VEGF production is stimulated by hypoxia-induced transcription factors such as HIF-1α and HIF-2α, but both oxygen treatment and systemic diseases such as respiratory distress trigger VEGF production [76].

Although the role of VEGF is established in both ROP development and treatment, the role of VEGF receptors (VEGFR 1 and 2) is less clear. VEGFRs promote the differentiation of endothelial cells, are important in angiogenesis, and their expression is increased by hypoxia and potentiated by VEGF [77]. In a small study measuring plasma levels of VEGF and VEGF-receptor 1 and 2, only VEGFR2 was elevated in ROP patients (severe ROP was not analyzed separately) [78]. In another study, ROP was associated with an increase in VEGF and VEGF-R2 expression and blood vessel growth [79]. Conversely, in a model of OIR in rats, a VEGF-R2 inhibitor reduced intravitreal neovascularization [80].

#### Erythropoietin

EPO has multiple functions in the fetus and the newborn and was originally viewed as a hematopoietic hormone exclusively stimulating the production of erythrocytes [81]. Since then, other important functions of EPO have been identified i.e., related to the development of the brain, retina, cardiovascular [82], and gastrointestinal systems [83]. In preterm newborns, recombinant human EPO (rh-EPO) is used to reduce transfusion requirements [84] and appears to attenuate the risk of brain damage [85, 86], apparently through anti-inflammatory, anti-excitatory, and neuroproliferative pathways [87, 88]. Among preterm newborn, those who have elevated concentrations of endogenous EPO are more likely than others to have elevated concentrations of inflammation-related proteins in concurrent blood specimens [89]. Elevated EPO concentrations in the blood of infants born before gestation week 28 were associated with a variety of morbidities including ROP and respiratory problems occurring later [90]. It is possible that EPO plays a direct role in stimulating angiogenesis and is consistent with high EPO concentrations before ROP-associated neovascularization occurs [17, 21, 91].

EPO and its receptors are present in the retina, but the role of EPO in ROP pathogenesis remains to be clarified [84]. Meta-analyses report an increased risk of severe ROP with EPO-treatment [84]. Like VEGF, EPO is a potent angiogenic factor, and its production is induced by HIFs. It is conceivable that EPO plays different roles in the different phases of ROP development [92]. For instance, low concentrations of EPO in phase 1 contribute to a stop in angiogenesis, while elevated levels in phase 2 enhance pathological neovascularization. The concentration of EPO in the vitreous is correlated with that of VEGF and is elevated in the vasoproliferative phase of ROP [73].

#### Insulin-like growth factor 1

Insulin-like growth factor (IGF-1) is a hormone important for fetal growth, including healthy retinal angiogenesis [93]. IGF-1 is also probably necessary for normal VEGF function [94]. Importantly, the placenta and amniotic fluid are the main sources of IGF-1 during development in utero, such that after birth, IGF-1 levels decrease precipitously in premature infants [95, 96]. Inflammation is another factor that may further reduce the preterm newborn’s limited IGF-1 production [97]. Low systemic serum IGF-1 concentration is a risk factor of ROP development and is a biomarker that identifies infants at risk weeks before disease manifests [98]. Low IGF-1 serum levels are associated with retinal vessel growth delay, which is directly correlated with the severity of ROP [96]; interestingly, IGF-1 binding protein (IGFB3) was also found to be decreased in premature infants and may also contribute to retinal vessel depletion [96].

#### Placental growth factor

Placental growth factor (PlGF) is a protein in the vascular growth factor family and is upregulated in pathological angiogenesis. It is, presumably, an important cofactor for retinal neovascularization, but PlGF also plays a role in recruiting immune cells [99, 100]. Unlike its siblings in the VEGF-family, PlGF is downregulated during hypoxia and exerts an anti-apoptotic effect during hyperoxia. PIGF is therefore suggested to be important during the aberrant neovascular phase of ROP [101]. In a mouse retinopathy model, PlGF deficiency reduces pathological vascular leakage [102]. To add to this complexity, PlGF expression is increased upon treatment with anti-VEGF (bevacizumab) [99].

#### Basic fibroblast growth factor (bFGF / FGF-2)

Basic fibroblast growth factor (bFGF) is a potent stimulant of neuronal and endothelial proliferation and is expressed during vascular [103] and retinal [104] development. Akin to PlGF, expression of bFGF was downregulated when VEGF increases in cultured cells [105] and in mice with retinopathy [106]. Results from studies on bFGF are conflicting. A tendency of lower concentrations of bFGF was found in the vitreous of infants undergoing vitrectomy due to severe ROP compared to infants undergoing vitrectomy at term due to congenital cataract [13]. Increased retinal bFGF expression was detected in a mouse model of OIR [106], and retinal neuroprotective effects of bFGF are detected in various animal models [107, 108]. Still, other studies have reported that bFGF does not contribute to either the preservation of a healthy retina or to pathological neovascularization in animal models [109].

#### Angiopoietin 1 and 2

Ang1 and Ang2, vascular growth factors important both in fetal life and after birth, remodel the developing vasculature and contribute to vessel stability. Both are ligands of the Tie2 receptor, although in an agonist (Ang1) and antagonistic fashion (Ang2). When Tie2 and VEGF were inhibited together, retinal angiogenesis was more efficiently suppressed than with VEGF inhibition alone [110]. Whereas Ang1 promotes vascular maturation and stability, Ang2 works to initiate neovascularization [110, 111]. The concentration of Ang1 and Ang2 is negatively correlated in the vitreous of eyes with severe ROP, where Ang2 concentration was significantly increased [112]. These findings set the balance between Ang1 and Ang2 in ROP pathogenesis.

#### Thyroid-stimulating hormone

Thyroid function is essential for brain [113] and retinal [114] development. An association is found between neonatal hypothyroidism in preterm born children and poor neurodevelopmental outcome at 3 months and visual problems at 6 months [115]. However, in line with unclear definition of hypothyroidism in preterm infants, low T4 is not consistently associated with altered cognitive function compared to their euthyroid peers at age 7 [116]. When it comes to thyroid function and ROP pathogenesis, prophylactic supplementation of thyroid hormone has not reduced ROP prevalence [117]. Further on, hyperthyrotropin was associated with brain damage when occurring together with an inflammatory reaction [118], and it appears to contribute to both the onset and outcome of the inflammatory process [119]. Yet, both TSH and the thyroid function could be of interest in future ROP research.

### Semaphorins and ROP

The influence of neuron-derived signaling molecules on endothelial cell injury during ROP has recently been highlighted [44, 120, 121]. Classic neuronal guidance cues and their receptors, particularly class III semaphorins [122], a large family of conserved proteins originally implicated in axonal guidance, could act as repulsive molecules in the hypoxic area of the retina during retinopathy, thus hindering normal vascularization and contributing to the formation of abnormal vascular tufts [120]. The pathological mechanism that involve semaphorins in ROP is initiated when the resting microglia cells become activated in the ischemic areas of the retina and trigger the secretion of pro-inflammatory cytokines, particularly IL-1 that stimulate the production of pro-apoptotic/repulsive factor Semaphorin3A (Sema3A) specifically in retinal ganglion neurons [44, 120]. Augmentation of Sema3A in areas of ischemia then contributes to the vascular decay and forms a chemical barrier that repels neo-vessels towards the vitreous. Conversely, IL-1 receptor antagonist [44] or silencing Sema3A expression enhances normal vascular regeneration within the ischemic retina, thereby preserving neuroretinal function and diminishing aberrant pre-retinal neovascularization. Therefore, overcoming the chemical barrier erected by Sema3A accelerates the vascular regeneration of neural tissues, which restores metabolic supply, improves retinal function, and reduces the risk for abnormal intra-vitreal neovascularization.

In a similar manner to Sema3A, Wei et al. [123] recently showed that absence of neuronal Nrf2, a major stress-response transcription factor responsible for cell-intrinsic cytoprotective function, results in Sema6A induction in hypoxic/ischemic retinal ganglion cells that diminished normal revascularization into the avascular zones in the inner retina from ROP animals. Interestingly, lentiviral-mediated delivery of Sema6A small hairpin RNA (shRNA) abrogated the defective retinal revascularization in Nrf2-deficient mice. Importantly, pharmacologic Nrf2 activation promotes reparative angiogenesis and suppresses pathologic neovascularization [123]. These findings reveal a unique function of Nrf2 in reprogramming ischemic tissue towards neurovascular repair via Sema6A regulation, providing a potential therapeutic strategy for ischemic retinal diseases.

Other studies have also highlighted the anti-angiogenic properties of semaphorins. Fukushima [124] found that Sema3E expressed in retinal neurons guides normal and pathological vessels through its receptor Plexin-D1. In the OIR model, increased PlexinD1 expression in neovessels prevents VEGF-induced disoriented projections of endothelial filopodia. Of greater therapeutic significance, intravitreal administration of Sema3E suppressed pathological neovascularization, while preserving the desired regeneration of the retinal vasculature into ischemic retinal areas. The same effect occurs with the intravitreal administration of Sema3C. Yang [125] demonstrated that Sema3C acting through the receptors Neuropilin-1 and PlexinD1, which are strongly expressed on vascular tufts, induced VE-cadherin internalization and abrogated VEGF-induced activation of the kinases AKT, FAK, and p38MAPK. This resulted in a disrupted endothelial tip cell formation and cell–cell contacts that inhibited the formation of pathological pre-retinal vascular tufts during OIR. All these findings suggest that semaphorins are potential targets to be explored in the clinic setting to prevent ROP.

### Choroidal degeneration in ROP

At present, the inner retina has been considered the primary region affected in human ROP and animal models. However, it is known that dysfunction of the outer retina occur in a number of older children formerly afflicted with ROP [126–129]. Hence, with increasing survival of extremely premature neonates (at risk of ROP) [130], an insidious recognition of progressive outer retinal dysfunctions [126, 127] is being observed in former ROP patients, requiring follow-up beyond childhood [131, 132], as recently reviewed [133].

In recent years, several clinical and animal studies have reported extensive choroidal vascular degeneration associated with ROP development. Because choroidal vasculature is the exclusive source of oxygen and nutrients to the photoreceptors and RPE cells, it has been proposed that involution of the choroid may be associated with residual visual loss following ROP [35], as it leads to marked sub-retinal hypoxia [45]. In support of this inference, we have demonstrated that choroidal degeneration which occurs in the first postnatal week during OIR is followed weeks later by a loss in RPE integrity and a degeneration of photoreceptors [45]. Consistent with these observations, a number of recent clinical studies in older children and young adults revealed using optical coherence tomography a > 25% choroidal thinning in former ROP patients [132, 134–137]; importantly, this choroidal thinning is first detected in the human newborn [137], as documented in animal models of ROP [35]. Hence the vasculopathy seen in ROP affects not only the inner retina but also the choroid—important for RPE and photoreceptor integrity.

The mechanism of choroidal involution in ROP has been poorly explored. Shao et al. [35] demonstrated that a sustained degeneration of the choroid in different animal models of ROP is largely through the actions of the electrophile 15-deoxy-12,14-PGJ2 (15d-PGJ2), a nonenzymatic product of prostaglandin D2 (PGD2) that provokes apoptotic death in endothelial cells by inducing oxidant stress. Importantly, choroidal circulation in newborn animals is significantly controlled by high local levels of prostaglandin D2 (PGD2) [138, 139], which, in turn, curtails the autoregulation of choroidal blood flow in response to hyperoxic exposure, resulting in increased oxygenation of the outer retina [140]. Notably, a high density of PGD2 receptors is found in the choroid [141, 142]. The ensuing oxidative environment, as that seen in the immature subject [143, 144], facilitates the nonenzymatic conversion of PGD2 into 15d-PGJ2 [145], which participates in redox cell signaling [125, 146] and is cytotoxic to endothelial cells under high concentrations [147].

Zhou et al. recently [45] explored the role of IL-1β on choroidal involution and consequently on sub-retinal dysfunction in a ROP model in rodents. In this study, IL-1β was abundantly expressed in the sub-retina of OIR animals and was associated with an early-sustained involution of the choroid, which leads to a markedly hypoxic sub-retina and a progressive loss of RPE and photoreceptors. Early neonatal treatment with IL-1 receptor antagonist preserved choroid, prevented outer retinal hypoxia, and significantly blunted RPE and photoreceptor loss associated with OIR. These observations suggest a critical role for IL-1β (known to trigger prostaglandin synthase expression) in inducing choroidal degeneration and long-term sub- and outer- retinal disorder following OIR/ROP.

Interestingly, it has been noted that choriocapillary involution associated with OIR remains sustained into young adulthood, contrary to inner retinal revascularization that occurs in the middle of the second postnatal week. An additional mechanism may also involve VEGF which is increased in the inner retina during pathological neovascularization, but decreased in the choroids of rats subjected to OIR [35]. Accordingly, choroidal vasculature in preterm newborns continues to develop after exposure to lower oxygen levels [135]; however, high oxygen level exposition leads to less VEGF expression by RPE cells that may contribute to slowing choroidal development resulting in thinner choroid. A recent clinical study has proposed that the time of oxygen exposure is one of the most relevant and negative predictor for choroidal thickness in ROP patients [148]. Concordantly, persistent thin choroid in children with a history of ROP may also reflect a delay in choroidal development. Collectively, these findings highlight the importance and relevance not only of the retinal vasculature but also of that of the choroid as a possible contributor to adverse outcome of ROP.

In line with this concept, it should be pointed out that ROP patients are notorious to developing myopia later in life. Hence, choroid thinning may contribute. Yet in this context, the dioptric change in axial length is altered by a few millimeters [149], whereas thinning of the choroid in humans with ROP is in the range of less than 100 μm; hence, the choroidal contribution to myopia in ROP cannot be simply attributed to axial length changes, scleral growth, and dioptric shift of the outer laminal membrane should also be accounted for myopic changes in ROP [150].

### Current and potential therapeutics in ROP

#### Current treatment strategies—laser ablation and anti-VEGF therapy

As mentioned earlier, peripheral retinal ablation by conventional laser therapy has for many years been the gold standard of ROP treatment. Despite the fact that laser ablation substantially helps prevent blindness, it has moderate effects in eyes with posterior ROP [151], as it may permanently reduce the visual field in addition to inducing myopia [152]. Hence, the development of preventive and less destructive therapies for ROP is desirable. Accordingly, the potential use of anti-VEGF therapy in ROP has been proposed. The anti-VEGF bevacizumab was approved in 2004 by the FDA for treatment of metastatic colorectal cancer. Bevacizumab was shown to inhibit tumor angiogenesis and extend patients’ lives by about 5 months when given intravenously as a combination treatment along with standard chemotherapy drugs [153, 154]. Off-label use of bevacizumab for ocular neovascular diseases started shortly thereafter. In eyes with ROP, it was found to suppress pathological neovascularization in phase 2 and an RCT reported in 2011 benefit from bevacizumab injections compared with laser therapy in eyes with the most posterior ROP (in zone 1). Several clinical studies have documented the use of bevacizumab, ranibizumab, pegaptanib, and recently aflibercept as a therapy for the treatment of ROP [75, 155, 156]. A generally favorable outcome has been detected in the majority of the reports; however, in some of the studies, adverse effects such as endophthalmitis, hemorrhage, retinal detachment, and cataract were also reported [75, 155, 157]. Intravitreal injections of anti-VEGF are by many ophthalmologists considered the primary treatment in eyes with posterior ROP [158, 159]. The main advantages of anti-VEGF therapy over the use of laser include (a) easier administration under topical anesthesia [160–163], (b) less distortion of macula in posterior ROP [160], and (c) preservation of the peripheral retina that allows continuous vascularization of the retina and prevents the peripheral visual field defects of laser ablation [155]. Concerns have, however, been raised regarding the possible ocular and systemic effects of anti-VEGF in the premature infants [164]. Possible disadvantages during the use of anti-VEGF in the clinic or animals models include (a) systemic complications after intravitreal injections [155], (b) delayed vascularization of retina [160], (c) VEGF blockade alone cannot completely eliminate pathological angiogenesis, suggesting that other factors could be involved [165], and (d) the suppression of VEGF in the eye can affect the survival and/or the function of non-vascular cells including neurons [166], Muller cells [167], cilliary body [168], RPE cells, choriocapillaris, and photoreceptors [169]; this aspect is particularly relevant to the developing neonate in phase of troubling follow-up results in patients treated with bevacizumab [170]. Therefore, systematic research to maximize effectiveness while minimizing side effects of anti-VEGF therapy in ROP is still needed, as dose, duration of action, and molecular entity of anti-VEGF need to be considered.

### Potential preventive strategies

Some promising preventive approaches such as supplementation with IGF-1 and omega-3 fatty acids to prevent vascular injury in phase 1 ROP will be discussed below. Likewise, future therapies such as stem cell transplantation and gene therapies may be part of new strategies to treat ROP as well as targeted modulations of the inflammatory response in the preterm born infant.

#### Serum IGF-1

Consistent with the evidence that IGF-1 is as strong as predictor of ROP [98], Löfqvist, C. et al. proposed an algorithm named WINROP (weight, insulin-like growth factor I, neonatal, retinopathy of prematurity) in which IGF-I values can be used to screen for infants who might be at risk of ROP [12]. Using serial weight and IGF-1 measurements in a cohort of 50 premature babies, WINROP predicted all infants who later developed ROP by a mean age of 10 weeks [171]; but in other studies, prediction was not perfect, still requiring routine ophthalmologic evaluation based on current standards [172].

Nonetheless, IGF-1 supplementation has begun to be explored in premature babies at risk of ROP. Evidence to support such a trial has been backed by the following observations. Breast feeding increases serum IGF-1 [173]; this may be due to high levels of IGF-1 in human milk and/or to the presence of specific proteolysis of IGF/IGFBP-2 complexes that increase the bioavailability of IGF-1 [174]. In other studies, early fresh-frozen plasma transfusion, as a source of exogenous IGF-1, increased the serum levels of IGF-1 in premature infants and decreased the risk of ROP [175, 176]. Likewise, administration of rhIGF-1 in mice attenuated OIR [177]. Recently, a randomized, multicenter (phase IIb) clinical trial of continuous infusion of rhIGF-1 has been launched to address whether maintaining normal physiological levels of IGF-1 may prevent ROP (and other neonatal complications) in premature infants (NCT01096784).

#### Omega-3 supplements

Polyunsaturated fatty acids (PUFAs) such as docosahexaenoic acid (DHA) and arachidonic acid are fundamental structural components of neuronal and endothelial cells [178] and are required to maintain optimal retinal functions. Connor et al. [179] demonstrated that dietary omega-3 fatty acids protect against pathologic neovascularization in a mouse model of ROP. The protective effect of omega-3-PUFAs and their bioactive metabolites was mediated, in part, through suppression of the pro-inflammatory cytokine tumor necrosis factor-alpha (TNF-α) present in a subset of microglia that were closely associated with retinal vessels. These findings suggested the possibility that omega-3 fatty acid supplementation to premature infants may be of benefit in preventing ROP. In this regard, a recent meta-analysis showed that LCPUFA supplementation of infant formulas improves infants’ visual acuity up to 12 months of age [180]. However, the impact of fortification of infant formula or breast-feeding mothers supplemented with omega-3 fatty acids to treat ROP needs further study. Currently, the University of California is conducting a trial to supplement very low birth weight infants with omega-3 PUFAs for the prevention of ROP (NCT02486042).

### Future therapies

#### Stem cell therapy

Stem cells provide an attractive therapeutic approach in treating ischemic retinopathies including ROP, due to its potential in tissue regeneration. Several groups have shown that different populations of stem cells could promote vascular repair in OIR model [181–183]. For instance, myeloid progenitors were able to migrate to the retinal avascular areas, differentiate into microglia, and participate in the reduction of vasoobliteration and neovascularization in OIR-transplanted animals [181]. Furthermore, bone marrow-derived stem cells targeted retinal astrocytes and promoted or inhibited retinal angiogenesis [182]. Another candidate in cell-based therapy for ROP is endothelial progenitor cells (EPCs) [184]. Interestingly, EPCs derived from human peripheral blood (injected intravitreally) were found to incorporate in the resident vasculature and promote tube formation—essential for vascular remodeling in ischemic regions [185]. However, the benefits of EPCs remain controversial since they have also been shown to contribute to pathological neovascularization in humans [186, 187].

#### Gene therapy

Gene therapy may be a possible treatment option for patients with ROP. Viral vectors can be used to transport therapeutic material to cells of the retina [188]. Adenovirus (Ad), adeno-associated virus (AAV), and lentivirus are most commonly used for retinal transgene delivery [189]. In this regard, a recent study showed that sub-retinal injection of an AAV carrying any of a variety of antiangiogenic genes including endostatin, pigment epithelium-derived factor (PEDF), and tissue inhibitor of metalloproteinases 3 (TIMP3) was able to significantly inhibit pathological neovascularization in the ROP model [190]. Thus, gene therapy-mediated regulation of cytokines and growth factors involved in ROP is a potential albeit at present far-fetching approach in premature babies.

## Conclusion

**Fig. 1:**
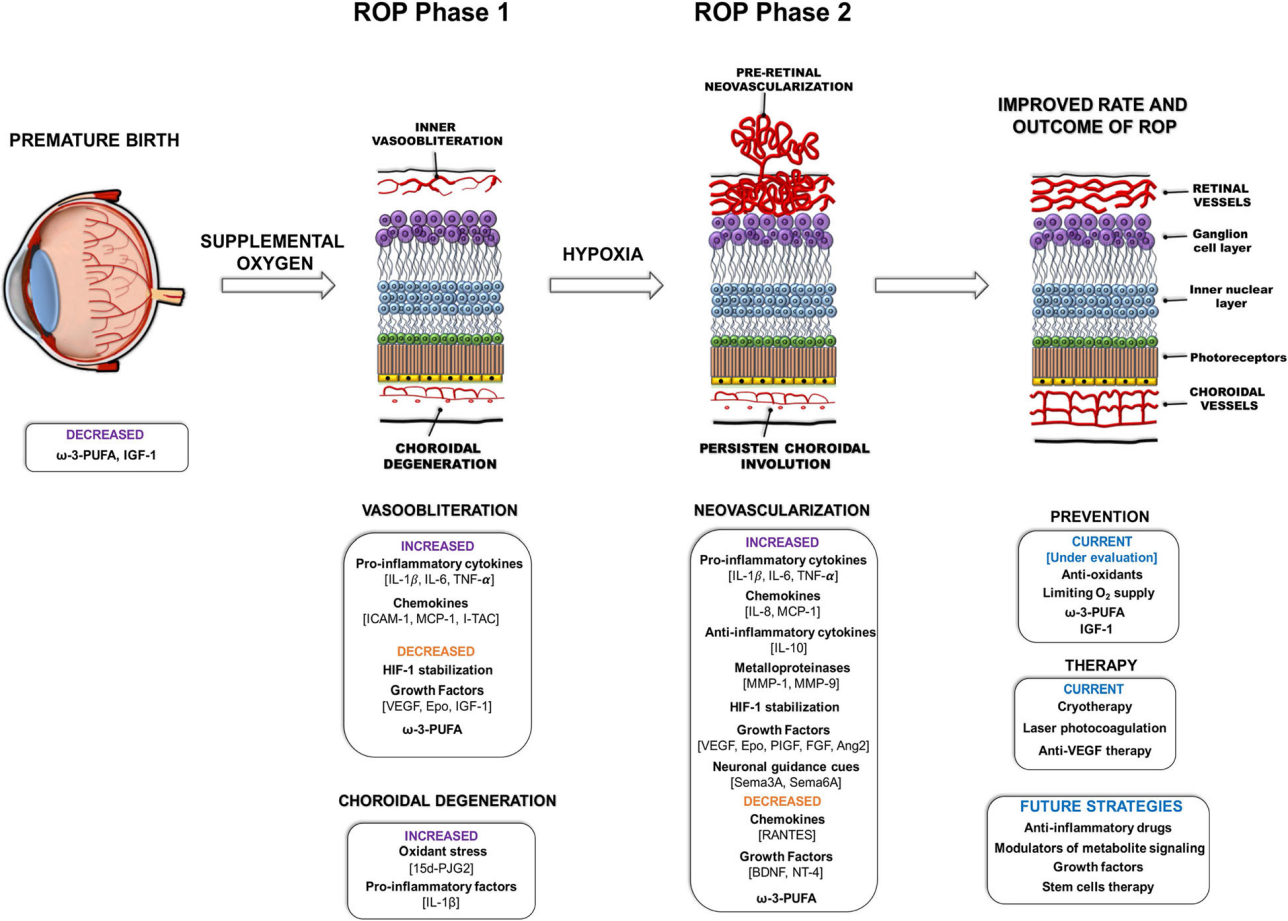
Summary of the current inflammatory and neuronal-derived factors involved in the pathogenesis of ROP. At birth, premature infants are deficient in factors essential for healthy blood vessel development. When premature infants are exposed to excess supplemental oxygen, the latter contributes to retinal and choroidal vascular obliteration due to oxidant stress, suppression of oxygen-regulated pro-angiogenic factors, and an excessive production of pro-inflammatory factors. Because of the vascular dropout, a compensatory, albeit aberrant and destructive, neovascularization occurs, driven by hypoxia-induced angiogenic factors. Some of the current therapeutic interventions rely on invasive procedures, such as laser photocoagulation, whereby affected areas of the retina are cauterized. Other treatments, including anti-VEGF therapy, as well as IGF-1 and omega-3, are currently being more thoroughly evaluated. In addition, the development of anti-inflammatory drugs as well as, future regenerative therapeutic interventions involving stem cells are also being explored and considered for the treatment of ROP

The search for biomarkers to identify children at risk of vision-threatening ROP has led to identification of several promising indicators related to inflammation and angiogenesis. Although many of the harmful effects of pre- and postnatal inflammation are known, there is a lack of validated markers that can help identify children at-risk of ROP. The two-phase development of ROP underscores the importance of assessing biomarker concentrations over time when studying ROP pathogenesis, since a specific biomarker can exert opposing outcomes depending on the disease phase. Less destructive tissue-preserving therapies (than current laser ablation) are being explored, and include IGF-1, PUFAs, and potentially stem cell therapy, as well as, modulation of factors involved in neuronal guidance of vasculature and inflammation. In the overall context of ROP, the role of choroid degeneration in ROP also needs to be accounted for (Fig. 1). Although it is clear that a large body of our knowledge on the molecular aspects of the pathogenesis of ROP stems from animal models with OIR, yet OIR does not fully reflect the complex human condition of ROP. This emphasizes the added importance of epidemiological studies with an unbiased molecular dimension.

## Abbreviations

15d-PGJ2: Electrophile 15-deoxy-12,14-PGJ2
AAV: Adeno-associated virus
Ad: Adenovirus
Ang1: Angiopoietins 1
BDNF: Brain-derived neurotrophic factor
bFGF: Asic fibroblast growth factor
DHA: Docosahexaenoic acid
EPO: Erythropoietin
IGF-1: Insulin-like growth factor-1
I-TAC: Interferon-inducible T-cell α chemoattractant
MCP1: Monocyte chemotactic protein 1
MMP: Matrix Metalloproteinase
Nrf2: Transcription factor NF-E2-related factor 2
NT-4: Neurotropin 4
OIR: Oxygen-induced retinopathy
PGD2: Prostaglandin D2.
PIGF: Placental growth factor
PR: Photoreceptors
PUFAs: Polyunsaturated fatty acids
ROP: Retinopathy of prematurity
ROS: Reactive oxygen species
RPE: Retinal pigment epithelium
Sema3A: Semaphorin 3A
Sema3E: Semaphorin3E
shRNA: Small hairpin RNA
TIMP3: Tissue inhibitor of metalloproteinases 3
TSH: Thyroid-stimulating hormone
VEGF: Vascular endothelial growth factor
WINROP: Weight, insulin-like growth factor I, neonatal, retinopathy of prematurity
